# People Who Chose the Preventive Natural Bioenergetics (NB) COVID-19 Treatment Safely Experienced a Significant Reduction of COVID-19 Symptoms Compared to the General Population

**DOI:** 10.7759/cureus.16277

**Published:** 2021-07-09

**Authors:** Thibaud E d'Oultremont

**Affiliations:** 1 Research Department, Natural BioEnergetics, Worthington, USA

**Keywords:** covid-19, symptoms, frequency, severity, safe, effective, natural bioenergetics, treatment

## Abstract

Background and aim: The aim of this study is to assess if people who chose to receive the preventive Natural Bioenergetics (NB) COVID-19 treatment would experience safely a strong reduction in frequency and severity of COVID-19 major symptoms (fever, cough, and shortness of breath) compared to the general population.

Experimental procedure: The preventive NB COVID-19 treatment is a double acupuncture meridian-based procedure that primes the immune system using acupuncture points and specific substances and sounds on precise body locations. Four hundred and thirty-nine people from seven countries (Canada, USA, Mexico, UK, France, Israel, and Belgium) voluntarily received the non-invasive preventive NB treatment. Data used for this study have been gathered between April 2020 and December 2020. The severity of cases experienced by the general population was statistically compared with those of the 42 infected people of this study.

Results and Conclusion: Our analysis suggests the population who chose to receive the preventive NB COVID-19 treatment experienced a strong reduction in frequency and severity of the three major symptoms of COVID-19 (p<0.01) compared to the general population. Nobody in that population needed hospitalization, including the elderly, which can be interpreted as a very significant clinical improvement. Most people did not report any side effects. Only small side effects were reported.

## Introduction

Applying a tested method to a new problem

Natural Bioenergetics (NB) is a bioenergetic healing system that has been utilizing energy procedures with great success for more than 30 years. Many thousands of those treatments have been offered routinely and safely by NB specialists, including the two treatments that have been used in this study. Side effects of those two procedures are small, similar to detoxification symptoms. The aim of those two procedures is to prepare people’s bodies for an infection to reduce the frequency and severity of symptoms [1]. The first procedure prepares the body to deal appropriately with the infection, and the second one prepares the body for the long term.

Disease frequency and severity

COVID-19 disease severity ranges from asymptomatic or mild, to moderate, severe, and critical disease [2-4]. Severe and acute symptoms require at least medical attention or hospitalization, and acute patients require the Intensive Care Unit (ICU) [4].

The largest cohort study of more than 44,000 adults with COVID-19 from China showed that the greatest number of cases went into the mild-moderate category at 81%. Severe (14%) and critical (5%) diseases were rarer. The overall case fatality rate (CFR) that was reported in this study was 2.3%, and a CFR of 49% was noted among those patients with the critical disease [5].

The Canadian Epidemiological Report [2] estimates that 16% of Canadians diagnosed with COVID-19 have been hospitalized, and among those, 20% have been admitted to the ICU, and 19% required mechanical ventilation. These numbers are similar to those reported in the U.S.A. (19%) [6].

In Canada, among hospitalized cases, 74% reported one or more pre-existing conditions. Individuals of 60 years of age and older comprised 68% of hospital admissions including 64% of ICU admissions, and 96% of deaths [7-9]. Canadian data are similar to what is seen in other countries, indicating that older adults and those with some underlying medical conditions are at higher risks for severe or acute disease [8-10].

The frequency of fever varied among studies, ranging from 44% to 91% [3-11]. In a large cohort study in Europe, fever was present in 45.4% of cases. In China, the two largest studies reported fever in more than 80% of patients [12,13]. Since the early days of the pandemic, fever has been understood as the major symptom of the COVID-19 infection along with cough and shortness of breath.

In many publications, fever was reported as a body temperature of a minimum of 37.3 degrees Celsius and any higher temperature [11]. Overall, 89% of patients with COVID-19 had increased body temperature during hospitalization, and maximum body temperature of over 38 degrees Celsius was measured in just half of the patients (38.1-39ºC in 47% and more than 39ºC in 2%) [11,14].

Cough has been reported in many COVID-19 patients [10]. A large European study reported the frequency of cough at 63% [15]. In two large studies from China, the cough was reported to account for 49% [16] and 66% of cases [17]. In a systematic review of the literature, the cough was found, on average, to be present in approximately two-thirds of adult patients with the non-severe disease [10]. However, due to its variable frequency, the cough should not be considered a sensitive indicator for COVID-19 disease [2].

A recent systematic review and meta-analysis found shortness of breath present among 44% of people with severe and 6% of people with non-severe disease [7]. Two large studies reported shortness of breath to range from 1% to 8% of mild cases, compared to 33% of severe cases [14,15].

## Materials and methods

Data and statistical analysis

The intent of the study was to conduct a study on a broad and varied population that could choose the treatment. This study was limited by the fact that it was not possible to offer the treatment to homeless and hospitalized people, even though some of them might have chosen it. Four hundred and thirty-nine people from seven countries (Canada, USA, Mexico, UK, France, Israel, and Belgium) voluntarily received the non-invasive preventive NB COVID-19 treatment. Educations, lifestyles, religions, and socioeconomic levels are varied in this population.

We compared the frequency and severity of COVID-19 major symptoms (fever, cough, and shortness of breath) of infected untreated people (general population) with symptoms of infected treated people. An independent t-test is powerful and robust enough to compare the means of two independent sets of data. As it is the most appropriate statistical method to compare those two means, we were using it whenever possible. The Percent Improvement and Treatment Effectiveness calculations were added for some symptoms to offer other statistical methods that are commonly used to assess treatment effectiveness.

Preventive COVID-19 treatment

Treatment Procedure

Pathogen’s frequencies placed appropriately on the body while holding specific acupuncture points can prepare the body before infection to reduce potential symptoms. A sugar pellet containing those specific frequencies with strengths comparable to those of homeopathic remedies was used for the first part of the treatment. The sugar pellet was placed on CV6 point (Central Vessel meridian or Conception Vessel meridian, point 6) while touching endpoints of all meridians in a specific sequence1 or using tools providing the same effect. For the second part of the treatment, the specialist touched the large intestine and lung meridians endpoints on his/her body (or using tools providing the same effect) while playing a sound file over his/her thymus.

Virus Frequencies Used

The first part of the treatment was aimed at reducing at least fever, cough, and shortness of breath and involved using a pellet that contained the frequency of the virus (with a strength similar to a homeopathic strength). The specialist did not use any real substance, but only a pellet filled with three different kinds of proprietary frequencies related to Coronaviruses available on the market: “Coronavirus” from ISHA Quantum metaphysics of NLS, “COVID-19” from LifeWorks Potential, UK, and “COVID-19” from Natural BioEnergetics, MA, USA. The second part of the treatment was done to prepare the body for the long-term, using the Spooky2 COVID-19 virus (Wuhan-specific) sound file.

Data collection

Data were entered into the database for four consecutive months: demographic data, COVID-19 test result and severity of four symptoms (fever, productive cough, dry cough, and shortness of breath/dyspnea) on a scale ranging from 1 to 10 (10 being the most severe). Other symptoms were not considered. Responses given to symptoms are subjective as people may have a positive or negative prejudice toward the treatment.

COVID-19 infection determination

As COVID-19 tests were not easily available until late in the study, we determined the presence of the COVID-19 if three of its major symptoms (fever, cough, and shortness of breath/dyspnea) were present together or if the person was tested positive. Out of the 441 people who received the treatment, 28 people experienced the three symptoms (6.3%), and 13 (2.9%) were tested positive and asymptomatic. During the first month after treatment, out of 441 people, 33 had at least two mild symptoms, and among those, five were able to get tested and tested positive, while 24 experienced the three symptoms. During the second month, out of 308 people having reported, four had the three symptoms, one having tested positive, nine people had at least two symptoms. Out of 266 people having reported, six had at least two symptoms for the third month, one having tested positive. Out of 246 people having reported, one had at least two symptoms for the fourth month.

Symptoms

Fever

We used well-documented data from people hospitalized as quality data in the literature is not available for COVID-19 infected people not needing hospitalization. We tested for the difference between the means of fever of hospitalized people (generally high fever) in the literature and the fever of treated people (generally lower fever) in our study. Nobody in our study had to be hospitalized. So, if the difference were highly significant, it would indicate that the fever of the treated population was very significantly lower than that of hospitalized people.

Cough

Productive cough and dry cough are generally not distinguished in the literature. So, to compare our data with available ones, we used the mean of those two symptoms in our study to calculate the frequency of cough. We also calculated the Percent Improvement as a way of assessing clinical significance in comparing our data with the frequency in the general population.

Shortness of Breath

Although shortness of breath is the main alarming symptom leading to hospital admission, data about its severity are only categorized broadly in the literature. Our data being more detailed than that found in the literature, we could compare our results to determine if the double NB treatment reduced the severity of shortness of breath. The Percent Improvement could be determined as well.

Severity of Cases

It is difficult to appreciate subjectively the severity of one’s case. Computing major symptoms is a way to remediate that issue even though it cannot describe objectively each case. In any case, fortunately, it is easy to classify some facts objectively: people sent to the hospital, or an ICU or dying. As nobody in our study needed to be sent to a hospital, we could objectively index the populations when it comes to severe and acute cases.

The severity of cases for each person was computed from the highest index of any major symptom followed in this study multiplied by 0.8 so that the most severe symptom without hospitalization would be attributed an index of 8. In the case of hospitalization, an index higher than 8 was assigned for severe cases with hospitalization, an index of 9 or higher for acute cases (requiring ICU), and an index of 10 for death. Indexed below 8 are more subjective: no symptom cases have classified an index below 1, while mild cases could be assigned an index between 1 and below 5, and moderate cases, higher than 5 and below 8.

Comparison of cases between the general population and that of this study has been done for symptomatic people only as cases reported in studies only account for symptomatic cases.

Vaccine effectiveness (VE) is used when a study is carried out under typical field (i.e., less than perfectly controlled) conditions. VE is interpreted as the proportionate reduction in disease among the vaccinated group. So, a VE of 90% indicates a 90% reduction in disease occurrence among the vaccinated group or a 90% reduction from the number of cases you would expect if they have not been vaccinated [18]. We are using the same formula to assess the effectiveness of the NB treatment. The formula is: ([Symptoms (%) no treatment - Symptoms (%) with treatment]/symptoms (%) with treatment) x 100.

## Results

Age

**Figure 1:**
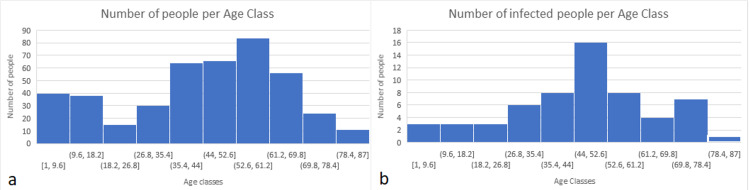
(a) Number of treated people per age class having received NB treatment. (b) Number of Infected people per age class in the study. NB - Natural Bioenergetics

The mean age for people having received the treatment is 42.1 (±22.2) years old, and 42.5 (±19.5) for those recorded as COVID-19 positive. Individuals having received the treatment for all ages comprise no hospital admissions, no ICU admissions, and no deaths. The highest percentage of infected people per age class in our study is 38%, for the 44 to 53 years of age. The percentage of infected people is about 11% for people above 60 years of age (Figure 1).

Fever

**Figure 2:**
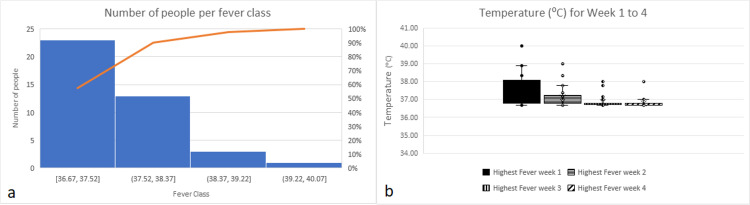
(a) Number of infected people per fever class during the first week. (b) Temperature of infected people for four consecutive weeks.

Among treated people, only one person had a high fever (Figure 2a) and no one had severe enough symptoms to have to be sent to the hospital. Also, our data show that the trend of fever decreases after the first week (Figure 2b).

**Figure 3:**
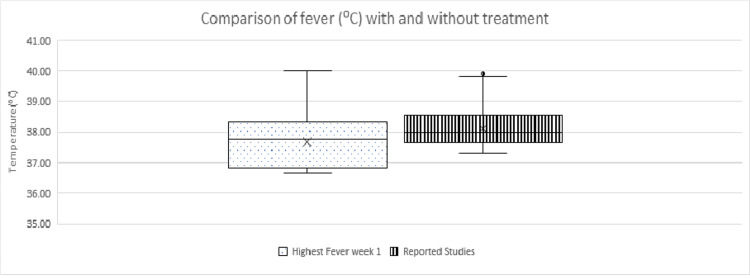
Comparison between fever in hospitalized patients in the general population and fever of infected people in our study during the first week.

An independent t-test with unequal variance was run on the data with a 99% confidence interval (CI) for the mean difference. It was found that the mean of fever with treatment (37.74ºC ±0.72) was significantly different from the mean of fever for hospitalized patients (38.14ºC ±0.29) (t(28)=2.46, p=0.02) (Figure 3). It is, therefore, possible to assume with confidence that people who chose to receive the preventive NB COVID-19 treatment experienced a reduction of fever compared to the general population.

Cough

**Figure 4:**
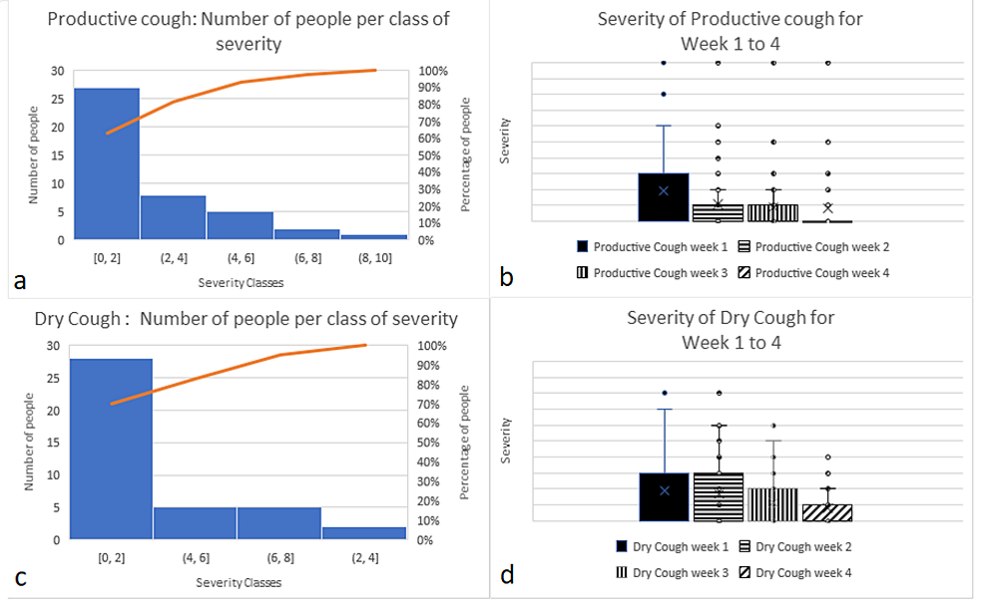
(a) Number of people with productive cough per severity class during the first week. (b) Severity of productive cough during four consecutive weeks. (c) Number of people with dry cough per severity class during the first week. (d) Severity of dry cough during four consecutive weeks.

The frequency of cough in our study is 37% for productive cough, and 30% for dry cough (Figures 4a, 4c). The Percent Improvement for the frequency of cough (mean of productive and dry cough = 33.5%) can be estimated between 32% and 49%. We noticed that the same person having had a 40ºC fever reported a productive cough with a severity of 10 for four weeks, and dry cough with the severity of at 8 for the two first weeks, and indexes of 6 and 4 for the two last weeks, respectively (Figures 4b, 4d).

Shortness of breath/dyspnea

**Figure 5:**
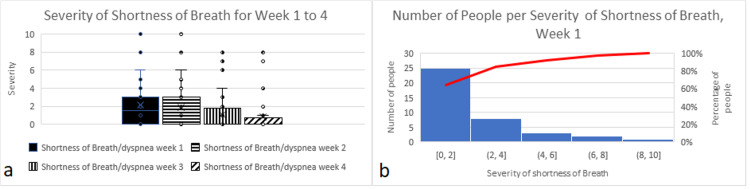
(a) Severity of shortness of breath during four consecutive weeks. (b) Number of people with shortness of breath/dyspnea per severity class during the first week.

The severity of shortness of breath is at its highest and almost identical during the first two weeks (Figures 5a, 5b). Our study shows asymptomatic to mild shortness of breath for 84.5% of treated people, moderate shortness of breath for about 13% of them, and severe shortness of breath for about 2.5% (which represents the same person having had high fever, high productive and dry cough) (Figure 5b). Importantly though, none of them was classified as severe or acute cases. In the general population, 33% to 44% of individuals severely infected by the virus experience shortness of breath, compared to 0% of treated people in our study. Consequently, the Percent Improvement of the NB treatments (33% to 44%) indicates a very significant clinical improvement of the frequency of shortness of breath.

Severity of cases

**Figure 6:**
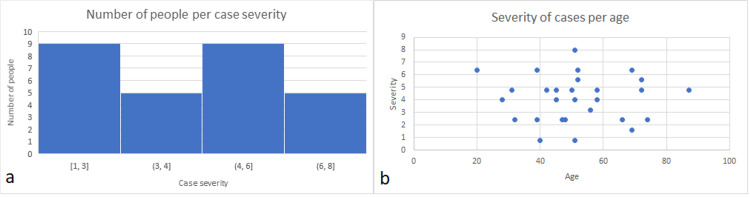
(a) Number of people per class of case severity during the first week. (b) Severity of cases per age.

Figure 6a shows the number of people per class of case severity for the first week during which the frequency and severity of all symptoms are the highest as compared with the other four weeks. As noted previously, there are no severe or acute cases reported in the study (nobody needed to be sent to the hospital), hence there are no cases with a severity index higher than 8.0. Asymptomatic people are not considered while comparing our data with published ones, as published data are lacking for asymptomatic people. In the general population, around 16% to19% of the population was sent to the hospital (severe and acute cases). The Percent Improvement in our study can therefore be estimated at around 16% to 19%. This indicates a high clinical significance in reducing the frequency of severe and acute cases. The effectiveness of the preventive COVID-19 treatment to avoid hospitalization is 100% as there are no severe or acute cases (no hospitalization) (Figure 6a). Also, the severity of symptoms does not seem to increase with higher age in our study (ANOVA significance of 0.92) (Figure 6b).

**Figure 7:**
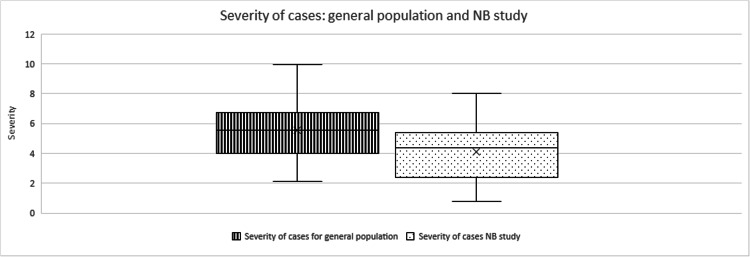
Severity of cases for the general population and the treated population of this study.

An independent t-test with unequal variance was run with normalized data from the general population, with a 99% CI. It was found that the mean of case severity (4.11±3.35) with the NB treatment was very significantly different from the mean of case severity for the general population (5.53±3.40) (t(43)=2.69, p=0.0007) (Figure 7). It is, therefore, possible to assume with high confidence that people who chose to be treated with the preventive COVID-19 treatment experienced a very significant reduction in the severity of the three major COVID-19 symptoms.

Safety of the NB treatment

Most of the 439 people did not experience side effects after treatment. A reaction close to a detoxification reaction occurred for less than 25% of people, and those side-effects varied greatly. Symptoms reported included feeling mildly “fluish,” headache, mild body soreness, and mild cough for one day. Most of the symptoms occurred within 24-48 hours. Four people reported those symptoms at 14 days without COVID-19 infection. No fevers were reported for people having been tested negative for COVID-19.

## Discussion

The aim of this study is to assess if people who chose to receive the preventive NB COVID-19 treatment would experience safely a strong reduction in frequency and severity of COVID-19 major symptoms (fever, cough, and shortness of breath) compared to the general population.

The independent t-test applied to the comparison of treated and non-treated people in terms of fever suggests a significant reduction of that symptom (p<0.05), while the same method applied to the three major COVID-19 symptoms suggests a very significant reduction in the frequency and the severity of those symptoms (p<0.01).

The frequency of both cough and shortness of breath has been reduced dramatically in the NB treated population compared to the non-treated one: between 32% and 49% for cough and between 33% and 44% for shortness of breath. Also, the frequency of those having to be sent to the hospital was 0% as compared to about 16% to 19% for the non-treated population. These results indicate a very significant clinical improvement in the NB-treated population.

This analysis also suggests that, in this study, the effectiveness of the preventive NB COVID-19 treatment to avoid hospitalization (severe and acute cases) is very high (100%). Individuals aged 60 or higher comprise about 68% of hospital admissions without treatment. With the NB treatment, that population had no hospital admissions. This high reduction of hospital admission for the elderly can also be interpreted as a very significant clinical improvement.

The preventive COVID-19 treatment was safe in this study. When side effects were present, they were not important.

The result of this study raises a question: How could the preventive NB COVID-19 treatment, using the frequency of the virus and acupoints, prepare the immune system for a COVID-19 infection, holding that preparation for at least several months?

Modern research has proposed a variety of different interpretations as to how memory might be stored in soft tissues, possibly involving other forms of information storage not exclusively processed neurologically [19]. At the level of the fascial cell, for example, not only memory but also the awareness of the mechano-metabolic information it feels are present, and it has the anticipatory predisposition in preparing itself for alteration of its natural environment [20]. The precise way quantum physics works to bring information through the body and in cells via microtubules-associated proteins, or DNA or other biological structures needs further research [21]. Our study seems to add one more piece of evidence to the increasing body of evidence that memory or information (in this case, memory or information related to a particular pathogen) can be stored in the body. More precisely, it seems that the cells receive though the preventive NB COVID-19 treatments an anticipatory predisposition in preparing themselves for the presence of the pathogen through an energy transformation, allowing the whole body to react to the pathogen in the most appropriate way it can, possibly bringing some side effects that might be different from person to person as the entire body’s energy will be somewhat transformed differently from person to person.

## Conclusions

Our analysis suggests the population who chose to receive the preventive NB COVID-19 treatment experienced a significant reduction in frequency and severity of the three major symptoms of COVID-19 compared to the general population. Nobody in that population needed hospitalization, including the elderly, which can be interpreted as a very significant clinical improvement. Most people did not report any side effects. Only small side effects were reported.

## Appendices

Appendix A. Supplementary data

Supplementary data to this article can be found online at http://dx.doi.org/10.17632/s6p598hdcz.1
